# When Is Resection of a Cardiac Tumor With Metastatic Disease Acceptable?

**DOI:** 10.1016/j.jaccas.2024.102981

**Published:** 2024-12-18

**Authors:** Nitish K. Dhingra, Abdulaziz M. Alhothali, Dambuza Nyamande, Abdullah H. Ghunaim, Robert J. Cusimano

**Affiliations:** Division of Cardiac Surgery, Toronto General Hospital, University of Toronto, Toronto, Ontario, Canada

**Keywords:** cardiac tumor, metastatic cancer, surgical resection

## Abstract

Metastatic disease is a relative contraindication for resection of malignant cardiac tumors. However, certain situations may present themselves when primary cardiac resection may be warranted. We present a 21-year-old male diagnosed with metastatic epithelioid hemangioendothelioma with right ventricular outflow tract involvement for whom surgical resection was successfully performed and discuss strategies.

## Graphical Abstract

**Figure:**
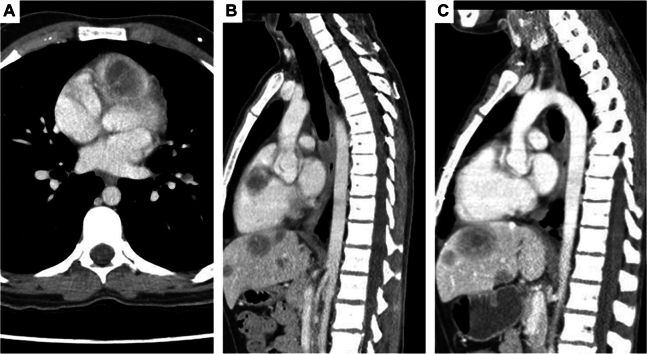

A 21-year-old male with known prior anaplastic ependymoma that had been diagnosed at the age of 2 (for which he had received multiple neurological resections and chemoradiation) underwent a routine surveillance echocardiogram and was found to have a large echogenic right ventricular outflow tract (RVOT) mass. The patient, who was asymptomatic and hemodynamically stable, underwent cardiac magnetic resonance imaging which demonstrated a 4.6-cm intracavitary mass subtotally obstructing the RVOT (Figures 1A and 1B). Unfortunately, extensive metastatic deposits in both lungs, hilar and mediastinal nodes, spleen, liver, kidney, and thoracic spine were noted. A liver biopsy yielded results consistent with epithelioid hemangioendothelioma^1^ with TFE3-YAP1 rearrangement. The patient was referred for cardiac surgical opinion, with an initial plan for conservative management given the lack of symptoms and the presumed lack of prognostic benefit in the setting of metastatic disease. The case was discussed at an international cardiac tumor board and it was determined that the pathology may have a long, protracted course and may be highly treatable. Given the subtotal RVOT obstruction, resection to prevent sudden cardiac death and to allow treatment for the metastatic disease was recommended. The patient’s mass, adjacent to the left anterior descending artery and in the free wall of the RVOT was resected with the RVOT, and his RVOT was reconstructed using bovine pericardium (our “go to” material for cardiac reconstruction). The patient subsequently received everolimus and, 3 years post-surgery, is clinically well with stable metastatic disease and no recurrence of the RVOT tumor (Figure 1C).

**Take-Home Messages**

• Resection of a cardiac tumor in the face of metastatic disease is not usually associated with improved outcome unless the lesion is imminently lethally obstructive, resectable and there is a treatment that will otherwise allow for reasonable response, and thus prognosis, for the metastatic disease.
• The metastatic disease in these situations must otherwise not be extensive enough to independently severely affect short-term survival.

**Figure 1:**
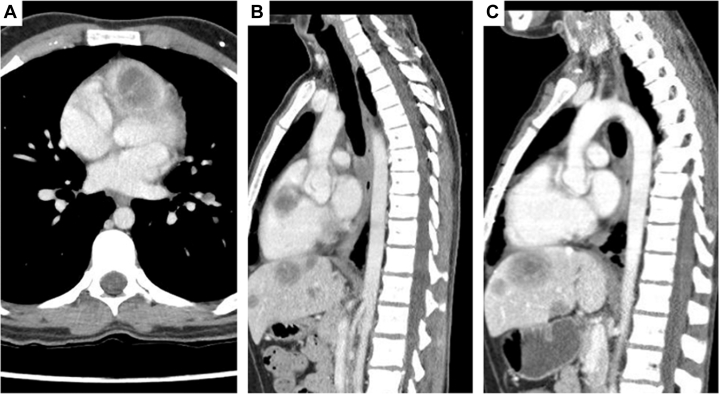
Serial Imaging of Epithelioid Hemangioendothelioma (A, B) Serial imaging of epithelioid hemangioendothelioma with initial preoperative imaging showing subtotal right ventricular outflow tract (RVOT) obstruction by the mass in the long- and short-axis. (C) At 3 years postoperatively, there is no recurrence of the RVOT tumor and relative stability of other metastatic lesions while on treatments.

The outcome of stage 4 cancer is poor. With multiple metastases, operation in isolation of one or even a group of lesions, leaving others, would not be expected to improve survival. However, when there is an expectation of reasonable outcome with subsequent treatment, and there is an operable lesion that will be imminently fatal, operation would be considered reasonable to allow further life-prolonging therapy. Without this proviso, operation is fruitless. There must be a reasonable surgical plan and an understanding that the operation alone is not curative. These individuals would continue their treatment regimens and, when undertaken, overall expectations for response and prognosis should be otherwise good for the patient to be able to recover from the operation and to enjoy a good amount of time after systemic treatment. If metastatic disease is so extensive or progressive, or treatments are not expected to be successful so that prognosis is otherwise poor and limiting, primary resection of the obstructing cardiac lesion is unwise and contraindicated.

## Funding Support and Author Disclosures

The authors have reported that they have no relationships relevant to the contents of this paper to disclose.
